# Effect of a daily outdoor access on milk quality and behavior of Italian Simmental dairy cows

**DOI:** 10.3389/fvets.2025.1659593

**Published:** 2025-11-17

**Authors:** L. Bailoni, S. Arango, N. Guzzo, S. Curró, N. Amalfitano, E. Bianco, E. Simonetti, S. Rainis, C. Sartori

**Affiliations:** 1 Department of Comparative Biomedicine and Food Science (BCA), University of Padua, Padua, Italy; 2 Department of Agronomy Food Natural Resources Animals and Environment (DAFNAE), University of Padua, Padua, Italy; 3 Regional Agency for the Rural Development (ERSA), Gorizia, Italy

**Keywords:** welfare, cattle management, external paddock, behavioral assessment, milk composition

## Abstract

This study aimed at assessing the effect of a two and a four-hour daily outdoor access on milk quality and behavior of dairy cows. Six Italian Simmental lactating cows housed in a free-stall were paired and subjected to the treatments: no outdoor access (CTR), two-hour daily outdoor access (U2; 11:30 a.m. to 1:30 p.m.), and four-hour daily outdoor access (U4) divided into a morning (9:00 to 11:00 a.m.) and an afternoon (2:00 to 4:00 p.m.) exit. Using a crossover design, each pair of cows was subjected to each treatment for 2 weeks, then switched twice, until the completion of 6 weeks of evaluation. Variations in milk parameters were determined across the treatments (CTR, U2, U4). Outside behaviors were assessed during the two-hour stay in the paddock, whereas inside behaviors were considered in the same timeslots plus an additional timeslot (4:00 p.m.–6:00 p.m.) in which the three treatments were inside the stall. Milk yield, composition and cheese-making traits were not affected by the outdoor access, but the coagulation properties were suggestively significant (*p* < 0.1). The longest rennet coagulation time (RCT, 21.31 min) and the lowest curd firmness (a30, 26.66 mm) were shown in U2. During the time spent outdoors, cows significantly increased their time standing resting (60.91 vs. 23.96 min; *p* < 0.001) and self grooming (6.58 vs. 2.96 min, *p* < 0.001); whereas, behaviors such as running, recumbency, drinking, eating, exploring and positive and negative interaction were reduced. When outdoors, cows spent most of their time standing resting (60.91 min), ruminating (11.10 min) and walking (10.62 min). Indoors, they spent more of their time eating (35.02 min), standing resting (23.96 min) and ruminating (13.84 min). Behaviors that were significantly affected by the treatment within each timeslot were: running, standing, resting, ruminating and eating. In conclusion, offering lactating dairy cows a four-hour daily outdoor access split into 2 h in the morning and 2 h in the afternoon appears to be beneficial because it increased the time spent standing resting outdoors which may indicate a calm state for the animals, while maintaining milk quality at a level comparable to that of full indoor management.

## 1 Introduction

Dairy production intensification has increased the use of indoor housing systems. Indoor confinement provides a controlled environment for cattle and assures good milk production if a high-energy feed is given. However, this type of housing can lead to negative impacts for the animals’ health such as problems related to hock lesions, mastitis and lameness (1–3). This is why new approaches using mixed housing systems have been developing (4–6). However, the provision of pasture can be difficult, especially for large intensive farms, and depends on the availability of land from the farmers (7). Moreover, some literature has argued that the pasture access may decrease the productive performance of animals because the feed is less balanced than that provided in farm (8). One possibility is to use an external paddock, not for pasture but just as an exercise area for the animals to stay during some hours with the benefit that it can be used the whole year round (9). This approach may provide cows the same benefits as pasture because they would be able to freely stand, walk and lie down. Access to an outside paddock allows cattle to express normal behaviors and provides comfortable lying space. Natural behaviors such as social interaction and environmental exploration are enhanced by providing outdoor access (3). Moreover, this type of cattle management is considered an option that meets public concern over animal welfare (10, 11). Most studies present this outdoor access as a choice (5, 6, 12, 13) rather than as a forced system, and this may also impact welfare in a different way. In addition, even though this seems a good alternative for dairy cattle, some aspects such as the best time of the day and the duration of the exit are still unknown.

The lactating period is an important stage not only for the animal but also for the farmers, as their entire profit can be made in this time. During this phase, cows increase their metabolic demands to produce milk and specific attention to their surroundings and husbandry practices is required to ensure the provision of a comfortable environment. The importance of giving outdoor access during the lactating period may be effective in improving welfare (3, 14) but we need to avoid affecting milk production. Around 80% of dairy cattle in Italy are housed indoors, and allowing a daily outdoor access can improve health and welfare in dairy cows. Currently, official legislation does not exist in this country but the possibility of spreading and regulating outdoor access of dairy cows is receiving an increasing amount of attention in scientific literature (3, 15). The aim of this study was to investigate if the provision of 2 and 4 h of daily outdoor access affects milk composition, cheese-making traits, milk coagulation properties, and the behavior pattern of Italian Simmental cows.

## 2 Materials and methods

This study was performed in accordance with the ethical committee of the University of Padova (approval number 36/2023) and carried out according to the directive 2010/63/UE of the European Parliament on the protection of animals used for scientific purposes and the Italian law on animal care (Legislative Decree No. 26 of 14 March 2014).

### 2.1 Animals

The entire herd was composed of 18 dairy cows that were raised and were kept in the stall. From this herd, six Italian Simmental lactating cows (DIM 103 ± 35, parity order 2.0 ± 1.4) were considered for the trial based on days in milk, parity, and the absence of mastitis and lameness. The animals did not have prior experience of outdoor exercise and the minimum statistical requirement was ensured to estimate data variability (16).

### 2.2 Breeding facility and management

**Figure 1:**
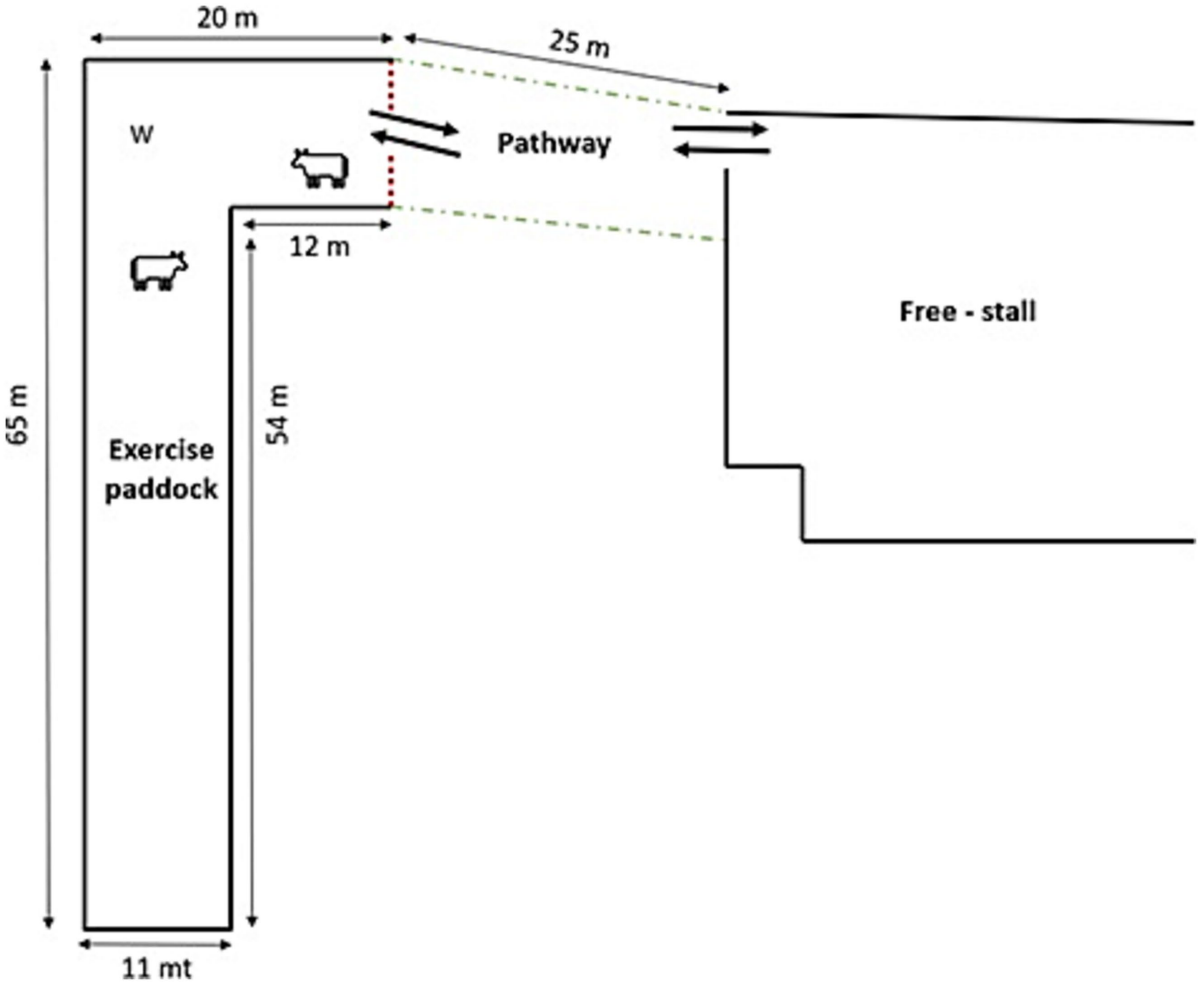
Schematic diagram of the stall and outdoor paddock. W, waterer.

The study was conducted at the dairy farm of the Institute of Higher Education (ISIS Paolino d’Aquileia) located in Cividale del Friuli (Udine, Italy). The breeding facility (Figure 1) consisted of a free-stall equipped with an automatic milking system (Lely Astronaut 2, Lely, Maassluis, Netherlands) and an outdoor paddock. The indoor area had concrete flooring, one long concentrate feeder, and 3 water dispensers. Cows were able to access the automatic milking system at any time when they were indoors. The walking distance from the barn to the exercise paddock was 25 m.

The outdoor area (Supplementary Figure S1), encompassing 800 m^2^ and surrounded by an electric fence, adhered to the space allowance established by the Royal Society for the Prevention of Cruelty to Animals (17). There were no feeders outside and one big plastic tank with fresh water was available. This paddock was covered by grass, but before the experimental period, the grass was trimmed (2 cm high approx.) to prevent grazing and no further agronomical interventions were necessary throughout the trial. Inside the outdoor area, there were four trees of a few meters in height that provided shade in some moments of the day. Access to the paddock took place 5 days a week (Monday to Friday). Before going to the external area, cows were driven to the milk station in order to be milked and prevent mastitis due to the accumulation of milk in the udder. Then, the animals were led gently toward the outdoor paddock.

### 2.3 Diet

In accordance with the guidelines outlined by the National Research Council (NRC, 2001), all animals were fed a total mixed ration (TMR) based on grass-silage and alfalfa hay (Table 1) and maintained a forage:concentrate ratio of 62:38 to fulfill energy requirements for lactating dairy cows. The TMR was distributed twice a day, during the morning (8:30 a.m.) and the afternoon (5:30 p.m.). TMR samples were collected at the beginning and the end of the experiment, and then analyzed using near-infrared spectroscopy (NIRs) with a DS 2500 FOSS instrument (FOSS DS 2500, Foss Analytical, Hileroed, Denmark; spectral range 850–2,500 nm, reflectance mode). Additional compound feed was available, depending on daily milk yield, during the access to the automatic milking system of the farm. Fresh water was available ad libitum, both inside and outside.

**Table 1:** Ingredients and chemical composition of the total mixed ration^1^.

Ingredients, % DM	
Grass silage	26.4
Alfalfa hay	32.8
Barley straw	2.7
Corn meal	17.3
Barley meal	12.4
Compound feed^2^	8.4
Chemical composition, % DM	
DM, % as fed	51.2
Crude Protein	14.7
Ash	8.6
Lipids	2.0
NDF	38.7
ADF	24.7
Lignin	3.8
Starch	21.1
NE_L,_ MJ/kg of DM^3^	5.8

1 TMR, Total mixed ration provided by the automatic feeder. The amount of the compound feed available during milking is excluded.

2 Chemical composition of compound feed: Moisture 13.00%, Crude Protein 18.50%, Lipids 3.20%, Crude Fiber 6.10%, Sodium 0.43%, Ash 7.40%.

3 According to NRC (2001).

### 2.4 Experimental design

The cows were divided into three fixed pairs, which were alternatively assigned to the following treatments: (i) no daily outdoor exit (CTR); (ii) a two-hour daily exit (U2); (iii) a four-hour daily exit (U4). The CTR group stayed the whole day inside the free-stall with the rest of the herd, the U2 group had a midday daily outdoor access (from 11:30 a.m. to 1:30 p.m.), and the U4 group had first a morning outdoor exit (from 9:00 to 11:00 a.m.) and then an afternoon outdoor exit (from 2:00 to 4:00 p.m.). The 3 weeks before the experiment began were considered as an adjustment period for the cows to get used to moving in and out of the paddock and no data were recorded. The experimental period had a duration of 6 weeks divided into three periods of 2 weeks. Using a crossover design, each pair of cows was subjected to each treatment for 2 weeks, then switched, until the completion of 2 weeks of evaluation in order to ensure the three different group combinations.

The experiment took place from October 31 to December 7, 2022, during the winter season. Local climatic characteristics during those days were taken from the closest climatic station. The temperatures varied from 3.5 to 21.0 °C, with an average of 10.6 °C. The mean daily wind speed was 12.4 km/h and the maximum wind speed recorded was 32.2 km/h.

### 2.5 Milk sampling and composition

Milk samples were collected individually after the morning milking on the last day of each experimental period. All samples were transported, maintained at 4 °C and analyzed the day after at the Milk Laboratory of the Department of Agronomy, Food and Natural Resources, Animals and Environment – DAFNAE (University of Padova, Legnaro, Italy). Fat, lactose, protein, total solids, casein and urea content in milk were estimated using a MilkoScan FT6000 (Foss Electric A/S), following the procedures (18). Milk pH was measured using a Titralab AT1000 series with PHC805 (Hach Company, Loveland, Colorado, United States).

Individual cheese-making was performed to measure cheese yield (CY) and milk nutrient recoveries in the curd (REC) traits based on the procedure of Stocco et al. (19, 20). Cheese-making traits were calculated from the weights of the milk and the whey (in grams) and their chemical composition, as described in previous studies (21, 22). In brief, the target traits were CYcheese, CYcurd, CYsolids, and CYwater, expressed as the ratio of the weight (g) of each cheese wheel after ripening, fresh curd, curd solids, and curd water, respectively, in relation to the weight of the processed milk (g). In addition, RECprotein, RECfat, and RECsolids were determined as the ratio of the weight (g) of the protein, fat, and solids in the curd to the corresponding weight in milk (g). Recovery of energy in the curd (RECenergy) was calculated as the percentage ratio of the energy in the curd to that in the processed milk.

Milk coagulation properties of each individual milk sample were measured in duplicate, using two lactodynamographs (2 Formagraph instruments; Foss Electric A/S, Hillerod, Denmark). A calibration of the pendula was made before each trial session. The analysis followed the method described by (23), and the traditional parameters of milk coagulation properties were considered: rennet coagulation time (RCT, min) from rennet addition to gelation; time interval between gelation and reaching a 20 mm curd firmness (k20, min); curd firmness at 30, 45, and 60 min after rennet addition (a30, a45, and a60, mm).

### 2.6 Behavioral observations

Before the experiment, all observers attended a 2-h training session conducted by a professor specialized in behavioral studies. Subsequently, they evaluated cows’ behavior both in real time (2 h) and from a video recording (2 h). Inter-observer reliability was assessed using the k coefficient (24), with all observers achieving a minimum k ≥ 0.7, indicating good agreement (25). The methodology for outdoor and indoor behavioral observations was different.

Observations of outdoor behavior (in the paddock) were carried out in real-time by 12 trained observers working in pairs chosen from students of the Institute. The observers remained outside the paddock maintaining a distance from the animals so as not to induce stress in them. Cows exited 5 days a week (10 days per period) although the observations were conducted twice a week totaling 4 days per period. Animals were distinguished by their unique coat color patches. Individual behaviors were recorded on paper as occurrences at each minute of observation, then reported in an Excel spreadsheet and summed to obtain the total time (min) that an individual cow spent on each behavior during the 2 h (120 min) spent in the outdoor area.

Behaviors inside the stall were monitored using six digital video cameras placed at the entrance of the barn and connected to a digital video recorder (H.264 Standalone Digital Video Recorder (DVR); Atlantis, Atlantis-land, MI, Italy) for continuous recording of behaviors. Individual cows were distinguished from the herd by their unique coat color patches. The recordings were analyzed by three trained observers, and the videos were randomly assigned to avoid any observer effect. Even though the trial was composed of 3 periods of 2 weeks each, only some days were considered for evaluation: 3 days of the first period, 4 days of the second period and 3 days of the third period. Then, only 4 timeslots of 2 h duration each were evaluated: morning (9:00 a.m.–11:00 a.m.), midday (11:30 a.m.–1:30 p.m.), early afternoon (2:00 p.m.–4:00 p.m.) and late afternoon (4:00 p.m.–6:00 p.m.). Behaviors were taken every 5 min of registration and reported in an Excel spreadsheet. Behaviors were expressed in minutes within each timeslot.

All behaviors considered inside and outside the stall are described in Table 2. For the final analysis, grazing was considered as eating behavior because the paddock was just used as an exercise area and cows were not able to effectively eat or graze. Also, allogrooming was merged with positive interaction.

**Table 2:** Ethogram with behaviors considered inside and outside the stall.

Behavior	Description	Inside	Outside
Walking	Displacement slowly from one location to another.	X	X
Running	Rapid movement with constant changes of direction inside the pen	X	X
Standing resting	Standing on four feet, inactive in a relaxed posture; head ‘not moving	X	X
Recumbency	Resting or sleeping with the legs curled under the body	X	X
Ruminating	Chewing motions of teeth while standing, moving or lying	X	X
Grazing	Moving the head toward the ground trying to bite pasture		X
Drinking	Drinking from the water tank or water dispenser	X	X
Eating	Ingesting feed from the feeder	X	
Defecating	Elimination of feces standing or moving	X	X
Urinating	Elimination of urine standing or moving	X	X
Exploring	Sniffing various parts of the stall, floor or surroundings	X	X
Self-grooming	A cow licking any part of itself	X	X
Allogrooming	Grooming and licking another individual using gentle gestures	X	X
Positive interaction	Staying beside another cow with affiliative postures such as sniffing, smelling and touching gently	X	X
Negative interaction	Aggressive actions toward others such as pushing and biting	X	X
Mounting	Jumping by lifting both forelegs onto the rump of another cow	X	X
Not visible	Not visible from the camera or hidden behind other cows	X	

### 2.7 Statistical analysis

Milk traits were analyzed using a general linear model analysis, (GLM procedure, SAS Institute Inc., Cary NC, 2014), differing only for a fixed effect related to the different instruments used for the analysis. A mixed model was initially run on milk yield, but since the treatment did not have a significant effect on milk production, this analysis was no longer considered.

The model applied for milk composition and cheese-making traits was:

$$Y_{\text{ijklm}}=\mu +T_{i}+D_{j}+B_{k}+C_{l}+e_{\text{ijklm}}$$

where Y_ijklm_ is the target individual parameter, μ is the overall mean, T is the fixed effect of the treatment [three levels: no exit (CTR); two-hour exit (U2) and four-hour exit (U4)]; D is the fixed effect of the day of analysis corresponding to the last day of each experimental period (three levels), B is the fixed effect of the two baths used for the analysis; C is the fixed effect of the individual cow (six levels), and e_ijklm_ is the residual error.

Milk coagulation properties were analyzed using the following model:

$$Y_{\text{ijklm}}=\mu +T_{i}+D_{j}+L_{k}+C_{l}+e_{\text{ijklm}}$$

This model differed from the previous one only for the L effect, included instead of the B effect, that corresponds to the different location within the instrument used for the analysis (six locations in two instruments, for a total of 12 levels). In both models, a *p*-value < 0.05 was considered to determine statistical significance, whereas a *p*-value < 0.1 was indicated as a suggestive significance.

After some preliminary analysis considering the cow either as a fixed or as a random effect, behaviors included repeated individual observations, and were analyzed using a mixed model analysis (MIXED procedure, SAS Institute Inc., Cary NC, 2014):

$$Y_{\text{ijklmno}}=\mu +S_{i}+\mathrm{TT}:S_{\mathrm{ij}}+G_{k}+P_{l}+D:P_{\mathrm{lm}}+C:G_{\mathrm{kn}}+e_{\text{ijklmno}}$$

where Y_ijklmno_ is the target individual parameter, μ is the overall mean, S is the fixed effect of the environmental setting of the cows, with i that is either indoor or outdoor; TT is the combined fixed effect of the treatment-timeslot (three treatments: no exit (CTR); two-hour exit (U2) and four-hour exit (U4) by four timeslots, for a total of j = twelve levels) within the environmental setting; G is the effect of the group (three levels); P is the fixed effect of the experimental period (three levels); D is the fixed effect of the day of observation within period, representing the repetition of the sampling; C is the random effect of the cow within its group (six levels), and e_ijklmno_ is the residual error. The least square means for the levels of the S and TT effects were calculated and compared using a Student t test with a Tukey correction. The contrasts between the levels of TT were also calculated to compare the levels of the treatment effect within each timeslot. Again a *p*-value < 0.05 was considered to assess the statistical significance, whereas a p-value < 0.1 was reported as a suggestive significance.

## 3 Results

### 3.1 Effect of the outdoor access on milk quality

The descriptive statistics of the milk composition, cheese-making traits and milk coagulation properties are presented in Table 3. On average, cows produced 26.81 kg of milk per day, while fat and protein were 3.87 and 3.68%, respectively. Mean values of lactose and total solids were 4.90 and 12.87%, respectively. Coagulation parameters such as RCT, k20, a30, a45 and a60 were on average 19.94 min, 5.01 min, 31.37 mm, 42.33 mm and 42.24 mm, respectively. In addition, the variation (CV) in milk coagulation properties was higher (0.31 on average) than the variation values of milk composition and cheese-making traits. Among these, the greatest variation was in fat percentage (0.27) and the least variations were found in pH, lactose percentage, the percentage of solids without fat and RECprotein.

**Table 3:** Descriptive statistics of milk composition, cheese-making traits and milk coagulation properties.

Parameter	Mean	SD	MIN	MAX	CV
Milk production, kg/d	26.81	3.72	18.50	37.30	0.14
*Milk composition*					
pH	6.62	0.06	6.49	6.75	0.01
Fat, %	3.87	1.02	1.59	5.52	0.27
Protein, %	3.68	0.26	3.36	4.46	0.07
Lactose, %	4.90	0.09	4.76	5.06	0.02
Casein, %	2.77	0.19	2.46	3.28	0.07
Total solids, %	12.87	0.94	10.96	14.33	0.07
Solids not fat, %	9.23	0.26	8.94	9.88	0.03
Urea, mg/dL	39.08	4.34	31.24	47.41	0.11
*Cheese-making traits*					
CYcheese, %	9.34	0.96	7.38	10.90	0.08
CYcurd, %	13.71	1.32	10.81	15.45	0.10
CYsolids, %	5.93	0.67	4.42	6.91	0.10
CYwater, %	7.59	0.83	6.24	8.62	0.11
RECprotein, %	77.08	1.75	73.96	79.90	0.11
RECfat, %	75.62	5.22	64.22	82.68	0.02
RECsolids, %	45.94	2.46	40.33	50.09	0.07
RECenergy, %	60.17	2.97	54.51	66.10	0.05
*Milk coagulation properties*					
RCT, min	19.94	4.73	11.30	28.30	0.30
k20, min	5.01	1.76	2.45	10.15	0.24
a30, mm	31.37	13.88	5.08	54.30	0.35
a45, mm	42.33	9.51	20.632	60.60	0.44
a60, mm	42.24	13.25	8.08	61.76	0.22

SD, Standard Deviation. MIN, Minimum value. MAX, Maximum value. CV, Coefficient of variation.

**Figure 2:**
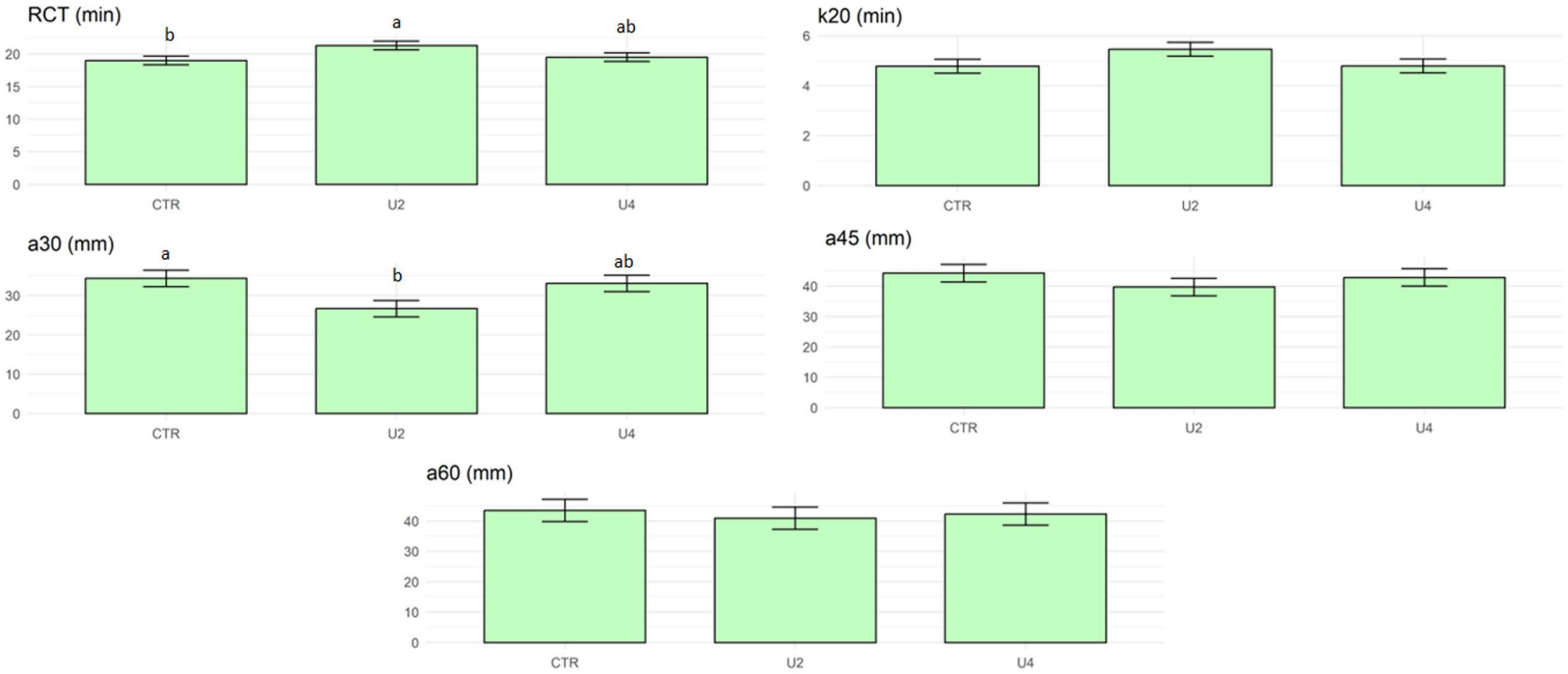
Least square means of the treatment effect on milk coagulation properties. Black lines represent SE.

The daily outdoor access did not affect milk composition and cheese-making traits, as reported in Table 4. The effect of the day of analysis resulted highly significant for pH and RECprotein, and suggestively significant for the urea content and CYwater. As expected, the effect of the bath was not significant and the suggestive significance for the lactose could be due to the low variability of this trait. The coefficient of determination of the model varied from 0.39 for CYsolids and RECsolids to 0.90 for pH. On the contrary, the daily outdoor access suggestively (*p* < 0.1) affected two of the milk coagulation properties, RCT and a30 (Table 5). Milk from cows with a two-hour daily outdoor access (U2) had a longer RCT (21.31 min) and a lower a30 (26.66 mm) than the CTR group. Meanwhile, U4 showed intermediate values for these two parameters (Figure 2). Additionally, the effect of the cow was highly significant (*p* < 0.001) for RCT, k20 and a30. The effect of the day was significant (*p* < 0.01) for all the traits. The location of the sample inside the instrument affected (*p* < 0.05) only k20. The coefficient of determination was high for all traits, especially for RCT, k20 and a30, with values equal to or above 0.90.

**Table 4:** ANOVA (*F*-value) of milk composition and cheese-making traits.

Parameter	Cow	Treatment	Day	Bath	R^2^
Milk composition					
Ph	3.042°	0.218	18.242**	2.594	0.90
Fat, %	0.610	0.374	0.608	0.073	0.44
Protein, %	0.564	0.104	0.576	0.154	0.41
Lactose. %	1.264	1.040	0.594	3.618°	0.67
Casein, %	0.595	0.200	0.240	0.223	0.41
Total solids, %	0.476	0.504	0.412	0.000	0.40
Solids not fat, %	0.420	0.458	0.182	0.764	0.54
Urea, mg/dL	1.581	0.002	4.181°	0.771	0.73
Cheese-making traits					
CYcheese, %	0.267	0.567	1.092	0.075	0.48
CYcurd, %	0.246	0.752	1.887	0.345	0.53
CYsolids, %	0.097	0.287	1.406	0.161	0.39
CYwater, %	0.689	1.221	3.688°	0.906	0.69
RECprotein, %	2.433	0.833	11.463**	0.017	0.86
RECfat, %	1.054	1.493	2.477	0.520	0.66
RECsolids, %	0.097	0.287	1.406	0.161	0.39
RECenergy, %	0.222	0.486	2.436	0.019	0.50

*p < 0.05. **p < 0.01. ***p < 0.001. °*p* < 0.1.

**Table 5:** ANOVA (F-value) of the milk coagulation properties.

Parameter	Cow	Treatment	Day	Location	R^2^
RCT, min	11.27***	2.87°	45.24***	1.25	0.92
k20, min	8.23***	1.64	23.93***	2.91*	0.90
a30, mm	9.73***	3.44°	39.68***	1.55	0.92
a45, mm	0.81	0.57	7.33**	0.82	0.66
a60, mm	0.23	0.10	14.79***	0.60	0.72

RCT, Rennet coagulation time. a30, a45, a60 = Curd firmness 30, 45 and 60 min after the addition of rennet. *p < 0.05. **p < 0.01. ***p < 0.001. °*p* < 0.1.

### 3.2 Effect of the outdoor access on behavior

**Figure 3:**
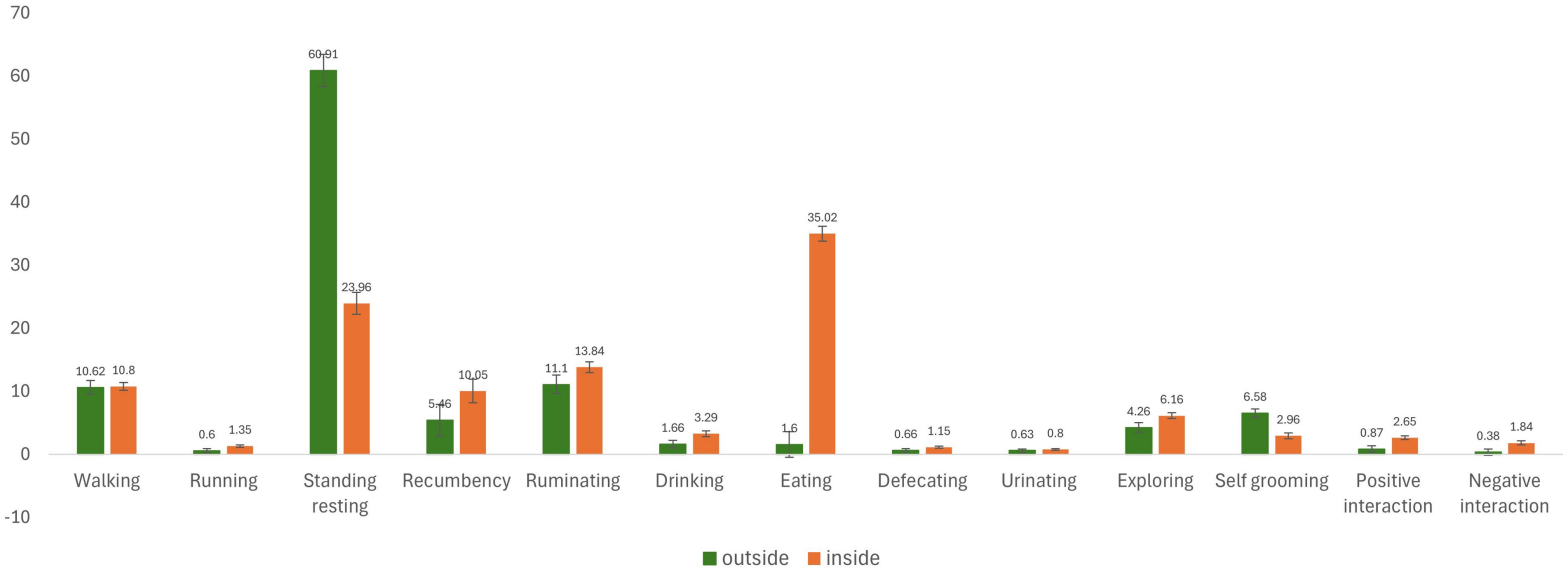
Least square means (min) of the setting (outside or inside) effect on behaviors in a 2-h timeslot. Black lines represent SE.

The ANOVA on fixed effects (Table 6) states that most behaviors were basically affected by the setting (inside or outside) and the day within period. The mean total duration of the behaviors observed during the two-hour timeslots inside the stall or outside (paddock) are shown in Figure 3. Nine of the behaviors showed statistical differences when animals were outdoors or indoors, detected considering the S term of the mixed model. Specifically, in comparison to indoors, the cows in the paddock increased their resting time (60.91 vs. 23.96 min; *p* < 0.001) and self grooming significantly (6.58 vs. 2.96 min; *p* < 0.001). On the contrary, other behaviors such as running, recumbency, drinking, eating, exploring, positive and negative interaction were reduced. When outdoors, cows generally spent most of their time standing resting (60.91 min), followed by other behaviors such as ruminating (11.10 min), and walking (10.62 min), whereas cows which remained indoors spent more time eating (35.02 min), standing resting (23.96 min) and ruminating (13.84 min).

**Table 6:** ANOVA (F-value) of the main fixed effects and interactions of all behaviors.

Behavior	Setting	Treatment-Timeslot	Group	Period	Day:Period
Walking	0.02	1.27	3.21	5.37**	4.26***
Running	3.58°	1.84°	0.13	6.79**	7.04***
Standing resting	196.98***	2.22*	1.65	5.61**	4.75***
Recumbency	3.72°	1.36	1.04	3.15*	1.93°
Ruminating	2.58	3.75***	1.95	1.86	2.31*
Drinking	7.77***	1.72°	0.61	3.82*	1.47
Grazing/Eating	203.22***	3.08**	2.83	4.65*	10.38***
Defecating	2.47	0.94	0.02	0.77	8.00***
Urinating	0.36	0.85	0.29	0.40	4.68***
Exploring	4.42*	0.99	1.78	8.44***	12.85***
Self-grooming	32.61***	1.40	3.46	2.50°	1.39
Positive interaction (inc. allogrooming)	10.84**	0.87	0.13	1.89	2.00°
Negative interaction	8.91**	1.50	0.92	0.05	2.09*
Mounting	1.47	1.03	0.49	1.96	3.75***

*p < 0.05. **p < 0.01. ***p < 0.001. °*p* < 0.1.

**Figure 4:**
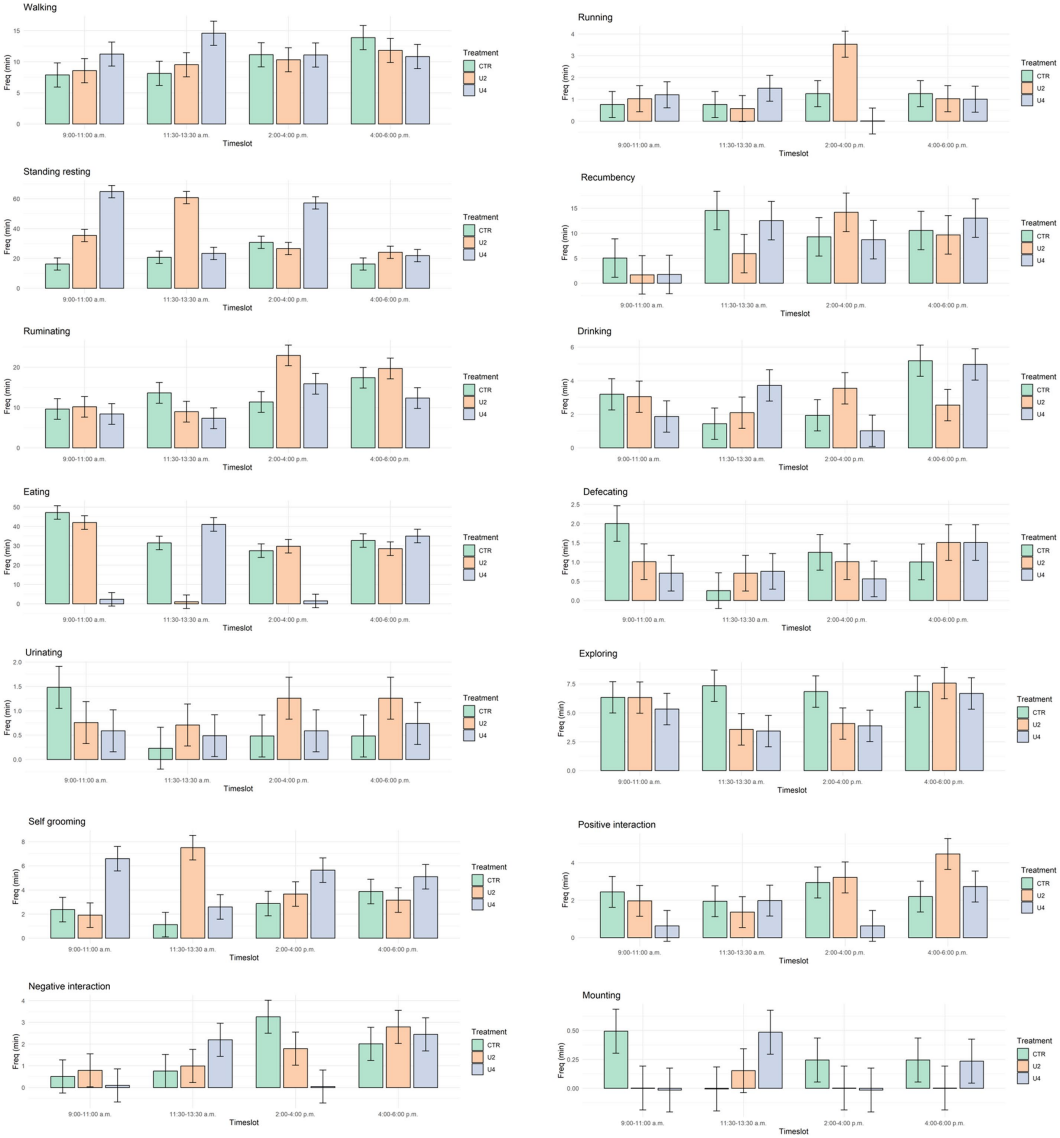
Least square means (min) of the behaviors considering the treatment effect within each timeslot. Black lines represent SE.

The duration of behaviors for each treatment within a particular timeslot is presented in Figure 4. Behaviors that were affected by the treatment within a timeslot were: running, standing resting, ruminating, drinking and eating (Table 6). The related orthogonal contrasts (Table 7) show that there were two main behaviors, standing resting and eating, that differed throughout the day.

**Table 7:** Orthogonal contrast (F-value) between the treatments and the timeslot effect.

Behavior	9:00–11:00 a.m.		11:30 a.m.–1:30 p.m.		2:00–4:00 p.m.	
	IN vs. OUT	CTR vs. U2	IN vs. OUT	CTR vs. U4	IN vs. OUT	CTR vs. U2
Walking	0.40	0.26	0.03	0.05	0.79	1.04
Running	15.36***	0.05	0.04	0.05	1.43	2.21
Standing resting	8.53**	51.19***	8.00**	52.93***	7.63**	53.62***
Recumbency	0.82	2.92°	0.67	2.46	0.44	0.13
Ruminating	13.74***	1.66	0.71	9.69**	8.79**	0.17
Drinking	2.76°	0.28	0.06	0.30	0.33	11.36***
Grazing/Eating	9.83**	37.39***	7.84**	27.74***	31.01***	39.50***
Defecating	0.86	0.48	0.06	1.48	0.00	0.46
Urinating	2.31	0.63	0.72	1.25	0.01	0.03
Exploring	0.70	3.86°	0.06	1.37	1.36	2.39
Self-grooming	0.32	22.55***	2.96°	6.51*	3.46°	1.73
Positive interaction (inc. allogrooming)	2.47	0.25	0.16	11.20***	0.33	1.86
Negative interaction	1.04	0.05	4.07*	6.73*	1.69	3.57°
Mounting	0.10	0.37	1.24	0.00	2.70	0.99

IN, inside. OUT, outside. CTR, no daily outdoor access. U2, two-hour daily outdoor access (from 11:30 a.m. to 1:30 p.m.). U4, four-hour daily outdoor access (from 9:00 a.m. to 11:00 a.m. and from 2:00 p.m. to 4:00 p.m.). *p < 0.05. **p < 0.01. ***p < 0.001. °*p* < 0.1.

## 4 Discussion

Discovering how the provision of 2 and 4 h of daily exit to dairy cows could influence milk composition, cheese-making traits, and milk coagulation properties is important, as it should not negatively impact the animal performance or the production parameters. At the same time, it is important to understand if and how much the behavioral patterns of the daily routine may differ between animals that had the possibility to spend or not some hours of daily exit in paddock. Moreover, the extent to which the duration (2 h or 4 h) of the management practice of providing an outdoor exit affects the behavioral patterns of the cows will also be examined.

### 4.1 Effect of the outdoor access on milk quality

As there was no effect of the treatment on any of the milk quality traits, breeders’ concern about the possibility of a negative effect on milk yield and quality from allowing dairy cows a daily outdoor access could be considered largely unwarranted. Daily milk production was not affected even though cows were outside 2 and 4 h a day without the possibility of accessing the milking robot. Knowing that allowing the cows to pasture in the management of high-yielding dairy cattle may result in them being unable to meet their nutritional needs (26), the use of an outdoor exercise area without pasture could be a better approach as in this trial. In addition, this type of management enables farmers to control the animals’ feeding since the outdoor area could not be grazed, preventing possible negative effects on performance. In fact, the absence of treatment effect on milk composition and cheese-making traits may support these observations even though some of the milk coagulation traits were slightly affected by one of the treatments. A detrimental effect was seen in cows with a two-hour daily access (U2) because of the longest RCT and the lowest a30. As the group of cows with a four-hour daily outdoor access (U4) had similar milk coagulation parameters to those without a daily exit (CTR), the combination of a morning plus an afternoon outdoor access could be considered as the best option for dairy cows. This raises an aspect of milking that should be considered. Even though cows were milked before exiting, it is possible that they would still have the urge to be milked during the time outdoors. When implementing an outdoor exit, an issue to consider would be how to deal with cows that have the need to reach the automatic milking machine while they are outside. Further studies could be carried out on this issue.

### 4.2 Effect of the outdoor access on behavior

There is still a lack of information regarding the use of an outdoor paddock in dairy farming and this is why some of the observed behaviors could not be compared to the literature. As was seen in this experiment, common habits of dairy cows are essentially diurnal (27, 28). Behaviors expressed inside the stall were apparently normal, as they were represented by the three main activities reported in the literature for dairy cows raised indoors: eating, standing resting and ruminating (29). After the provision of an outdoor access, changes in behavior of cows may occur not only during their outside stay but also when they go back inside to their normal free-stall housing (14).

When cows are provided a pasture access or offered a bigger space in an outside paddock, they are able to express greater locomotor activity (30). Normally, pasture access allows cows to graze, which involves movement. Grazing was not possible in this trial since the grass was trimmed, thus the amount of time spent walking outside did not increase in this trial. Indeed, the opposite effect occurred because cows were seen to run less when they were in the paddock.

Recumbency behavior as time spent lying is a highly motivated behavior in cows. This trial found no differences between the timeslots evaluated throughout the day, which can confirm the fact that cows tend to maintain their lying time (14). This could be seen in the time spent on recumbency by the CTR group of cows throughout the day. On the contrary, the effect of the setting showed that recumbency time was higher inside than outside. In fact, it was seen in the literature that cows replaced their lying time spent indoors with activity time when they moved to an exercise paddock (31).

The most evident behavior affected by the setting was standing resting. This behavior was found to be 2.5 times higher outdoors than indoors, changing the cows’ behavior pattern completely. During the two-hour period outdoors, dairy cows spent most of their time standing resting as the literature indicated (14, 29). This could be due to the fact that cows might find soil softer and more comfortable for standing than the hardness of the concrete flooring of the indoor barn. Moreover, cows spend more time standing outside when a higher space allowance is offered (14). Hence, passing from a low space allowance inside the stall to the paddock where each cow had a space of 400 m^2^ supports what was stated in the literature.

Ruminating is an important behavior for dairy cows. Even though the time spent ruminating was not affected by the setting, it represented the second and third most observed behavior outside and inside, respectively. The timeslot had an effect on this behavior as cows ruminate less during the morning and midday than in the early and late afternoon. The rumination pattern observed in the present experiment was similar to the diurnal pattern expressed by cows offered 24 h of pasture (38). The higher rumination time exhibited by cows during the afternoon timeslots reinforces the concept that rumination is modulated by feeding and time, because it is known that it tends to cease during and 2 h after feeding (32, 38).

Another noticeable behavior was drinking, as it is affected by several factors such as the housing conditions (32, 33). In fact, drinking turned out to be affected by the setting and the timeslot. Dairy cows in this study spent more time drinking inside than outside. This could be due to the number of waterers, the space allowance and the previous experience of cows. The NRC (2001) recommends 1 water bowl per 10 cows. Outside, there was one water bowl per 2 cows, whereas inside the stall there were three water dispensers. The space allowance could also be considered as a factor, as it was higher outside than inside. Finally, the fact that during their whole life these cows had been reared inside this stall might connect the drinking behavior to the type of water dispensers available inside the stall. This is why the waterer placed in the paddock might somehow have been considered as a “new device” and was not connected to their drinking activity. A difference between the timeslots was identified for the amount of time spent drinking, with the late afternoon being the timeslot in which cows used to drink more. A peculiar finding was that all groups of cows spent more time drinking 2 h post exiting and this, too, might be connected with the three factors previously mentioned.

In this study, eating was found to be the main activity of cows reared indoors. It is noteworthy that this behavior was practically excluded from the time budget of cows outdoors because the grass in the paddock was trimmed before the experiment, thus denying the possibility of eating or grazing. This could have resulted in a frustrated feeding behavior, as it was seen that cows sometimes tried to graze which was considered as eating behavior even though it was not effective eating. Reported data are scarce for grazing behavior inside this type of exercise paddock, indicating a low interest in the literature compared to other behaviors. Meanwhile, inside the stall, a clear difference in the distribution of the time spent eating throughout the day was found. Cows inside preferred to eat during the morning and at midday rather than in the early and late afternoon. This could be attributed to the time of feed distribution, which was around 8:30 a.m., half an hour before the morning timeslot (9:00–11:00 a.m.).

Grooming promotes health, calmness, well-being and overall performance (34). Regarding the duration of self-grooming, an experiment using Holstein dairy cows did not find differences between animals reared inside a stall and those with the possibility of using an external paddock (31). In this experiment however, cows using the paddock showed more of this behavior than those that remained inside the stall which could be considered positive.

Negative interactions, considered as antagonistic interactions, occur at low levels when animals pasture rather than when they are housed (2). In fact, our study showed an effect of the setting on the duration of this behavior. Even though cows did not have the possibility to pasture, the negative interactions inside the stall were almost five times greater than in the exercise paddock. A possible reason could be the stocking density inside the stall (35). Outside, cows were in pairs and there was little chance of their expressing negative interaction toward each other because of the large space allowance. Inside the stall, the experimental group of cows was surrounded by the entire herd, consequently they had more chances to express antagonistic interactions mostly because they were in a social setting in which sometimes aggressive behaviors arise in the search for food, water or simply because of hierarchy.

The space allowance affects cows’ behavior, especially locomotion activities, lying time and social interaction between them (14, 30). Cows offered more space outdoors are expected to express more locomotor activity (30), but such an effect was not found in this trial. The characteristics of the outdoor area (type of soil, type of grass, obstacles) can also have an impact on the extent to which animals feel comfortable expressing locomotion behaviors such as running or walking (30). It is possible that animals in this experiment found the paddock boring because it had no obstacles and no growing grass to motivate them to move around. Actually, a big part of the locomotor activity outdoors occurs on pasture where animals are able to graze and not with the use of these types of exercise yards (30). A bigger space reduces social interaction while a smaller space reduces lying times (14). In fact, the duration of behaviors that involved social interaction such as exploring and positive interaction was lower during the outside stay. Studies have examined space allowances of 3, 4.5, 9 and 16 m^2^ per cow but never one as large as the 400 m^2^ per cow used in the paddock of this trial. This allowance was established based on the current recommendation set by the RSPCA welfare standards for dairy cattle for open pens that has established that densities for paddocks can be a a maximum of 10 to 12 cows per acre (17).

Last but not least, the season of the year must be emphasized in the behavioral pattern observed in this trial. Dairy cows’ preference for an outdoor access is influenced by the weather (35), as they will avoid exiting with high temperatures and high light exposure and they tend to prefer to exit at night when temperatures are low. This trial was carried out during the winter season where temperatures ranged from minimums of 3.5 to 14.4 °C and maximums of 8.2 to 21.0 °C. Weather also influences the behavior pattern in dairy cows (36, 37). Therefore, cows may reflect a totally different behavior pattern if this experiment had been done during summer when temperatures in north Italy rise to 38 °C. In this case, cows might behave differently and may even refuse to go outside.

To conclude, the possibility of spending time outdoors significantly affected the individual time budget of lactating dairy cows without significantly influencing milk quality and cheese making parameters. A greater time spent standing resting during the stay in the paddock may indicate a calm state for the animals when outdoors. Our results suggest that providing lactating dairy cows with a four-hour daily outdoor access split into 2 h in the morning and 2 h in the afternoon could be beneficial in terms of well-being, without affecting milk quality and milk coagulation traits.

## Data Availability

The original contributions presented in the study are included in the article/Supplementary material, further inquiries can be directed to the corresponding author.
