# Supplementary figures and images for: Effect of a daily outdoor access on milk quality and behavior of Italian Simmental dairy cows
Source: Front Vet Sci. 2025 Nov 17;12:1659593. doi: 10.3389/fvets.2025.1659593 (PMC12665687; doi:10.3389/fvets.2025.1659593)

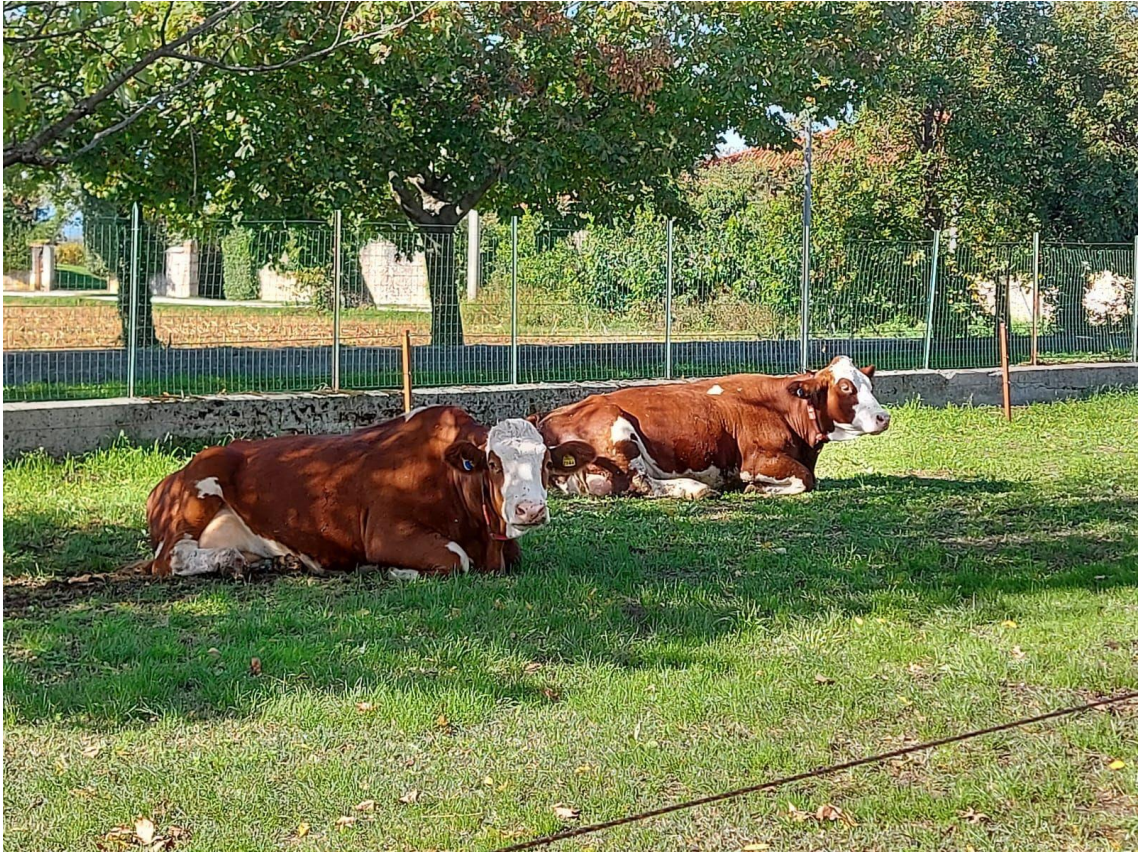

**Supplementary Figure 1.** Outdoor area used in the experiment.

Supplement: Supplementary file 1 [file Image_1.pdf]
